# Draft Genome Sequences of Four Methicillin-Resistant Staphylococcus aureus Strains (M080_2017, M106_2017, M181_2017, and M191_2017), Isolated from a Malaysian Teaching Hospital

**DOI:** 10.1128/mra.00867-22

**Published:** 2022-11-22

**Authors:** Mia Yang Ang, Nurul Amirah Binti Mohamad Farook, Norhidayah Kamarudin, Su Datt Lam, Sabrina Di Gregorio, Tengku Zetty Maztura Tengku Jamaluddin, Sheila Nathan, Muttaqillah Najihan Abdul Samat, Silvia Argimón, Hui-min Neoh

**Affiliations:** a Department of Gene Diagnostics and Therapeutics, National Center for Global Health and Medicine, Tokyo, Japan; b Department of Clinical Genome Informatics, Graduate School of Medicine, The University of Tokyo, Tokyo, Japan; c UKM Medical Molecular Biology Institute, Universiti Kebangsaan Malaysia, Kuala Lumpur, Malaysia; d Kulliyyah of Medicine, International Islamic University Malaysia, Gombak, Malaysia; e Department of Applied Physics, Faculty of Science and Technology, Universiti Kebangsaan Malaysia, Bangi, Malaysia; f Universidad de Buenos Aires, Facultad de Farmacia y Bioquímica, Instituto de Investigaciones en Bacteriología y Virología Molecular (IBaViM), Buenos Aires, Argentina; g Department of Medical Microbiology, Faculty of Medicine and Health Sciences, Universiti Putra Malaysia, Serdang, Malaysia; h Department of Biological Sciences and Biotechnology, Faculty of Science and Technology, Universiti Kebangsaan Malaysia, Bangi, Malaysia; i Department of Medical Microbiology and Immunology, Faculty of Medicine, Universiti Kebangsaan Malaysia, Kuala Lumpur, Malaysia; j Centre for Genomic Pathogen Surveillance, Big Data Institute, University of Oxford, Oxford, United Kingdom; University of Rochester School of Medicine and Dentistry

## Abstract

Draft genome sequences were obtained for four methicillin-resistant Staphylococcus aureus (MRSA) strains isolated from various wards of the Hospital Canselor Tuanku Muhriz (HCTM), Kuala Lumpur, Malaysia, in 2017. Using different bioinformatics tools, we annotated the draft genomes and identified multiple antimicrobial resistance genes.

## ANNOUNCEMENT

Methicillin-resistant Staphylococcus aureus (MRSA) is an important nosocomial pathogen. In this study, four MRSA strains isolated from Hospital Canselor Tuanku Muhriz (HCTM), a teaching hospital in Kuala Lumpur, Malaysia, in 2017 were selected for whole-genome sequencing and genome analysis. The study was reviewed and approved by the Research Ethics Committee of the National University of Malaysia (UKMPPI/111/8/JEP-2016-419); patient informed consent was waived by the committee. The strains were designated M080_2017, M106_2017, M181_2017, and M191_2017. M080_2017 and M106_2017 were isolated from blood, while M181_2017 and M191_2017 were isolated from sputum. M080_2017 was obtained from a patient with urosepsis, while the other three strains were recovered from pneumonia cases. The strains were identified as MRSA via Gram stain, colony morphology, DNase and tube coagulase positivity, and resistance to cefoxitin (Kirby-Bauer disk diffusion method) (1). The strains were grown in brain heart infusion broth (BD, NJ) at 37°C overnight prior to genomic DNA extraction using the DNeasy blood and tissue kit (Qiagen, MD).

Sequencing libraries were generated from genomic DNA using the NEBNext Ultra II DNA library prep kit. Whole-genome sequencing was performed using the Illumina NovaSeq 6000 sequencing system (2 × 150bp, paired-end). The raw sequencing reads were subjected to quality control, error correction, adapter trimming, and Q value determination via readfq v10 (https://github.com/billzt/readfq) using default parameters, where (i) elimination of low-quality reads (Q value, ≤38), (ii) removal of sequencing adapters, and (iii) filtration of duplicate reads were performed. Subsequently, base quality inspection was performed using fqcheck (2).

The clean reads were subjected to *de novo* assembly using a combination of SOAPdenovo v2.0.4 (3), SPAdes v3.15.4 (4), and ABySS v2.1.5 (5). The assembly results from the three different software programs were integrated using CISA (contig integrator for sequence assembly of bacterial genomes) (6), and the assembly with the least number of scaffolds was selected. GapCloser was used to fill any gaps within the preliminary assembled genomes. The quality of the assemblies were checked using the team’s internal scripts. The assembled genomes were annotated via the Rapid Annotations using Subsystems Technology (RAST) server (7). AMRFinderPlus v3.10 (8) was used to annotate antimicrobial resistance genes. Default parameters were used for all software. Genome assembly statistics and annotation features are shown in Table 1.

**TABLE 1:** Genome assembly and annotation statistics

Parameter	Data for strain:			
	M080_2017	M106_2017	M181_2017	M191_2017
Total no. of reads (bp)	3,337,351	3,564,167	3,136,578	3,096,992
Total assembly length (bp)	2,848,875	2,859,822	2,867,423	2,945,403
No. of contigs	36	21	40	34
GC content (%)	32.7	32.8	32.7	32.7
Shortest contig (bp)	1,051	1,563	609	1,223
Median contig length (bp)	54,674	111,107	40,192	54,817
Mean contig length (bp)	79,135	136,182	71,686	86,630
Longest contig (bp)	340,732	540,930	297,294	319,036
*N*_50_ value (bp)	156,500	234,863	146,478	156,497
No. of subsystems	274	272	275	175
No. of coding sequences	2,727	2,751	2,769	2,844
No. of RNAs (tRNAs, rRNAs)	62 (57, 5)	66 (57, 9)	65 (58, 7)	65 (57, 8)
Avg depth of sequencing coverage (×)	100	100	100	100

In conclusion, we report the draft genome sequences of four MRSA strains, M080_2017, M106_2017, M181_2017, and M191_2017. These new genomes may contribute further insight into the biology, evolution, diversity, and pathogenicity of this pathogen.

### Data availability.

The whole-genome shotgun project for strains M080_2017, M106_2017, M181_2017, and M191_2017 has been deposited at GenBank under the accession numbers JALLIS000000000, JALLIT000000000, JALLIU000000000, and JALLIV000000000 and the SRA accession numbers SRP394588, SRP394590, SRP394593, and SRP394595, respectively. The version described in this paper is the first version. The RAST annotation information is available at https://doi.org/10.5281/zenodo.7272980.
